# Extracts and Abstracts

**Published:** 1888-07

**Authors:** 


### EXTRACTS AND ABSTRACTS.

### THE UNSUITABILITY OF SILVER TUBES FOR TRACHEOTOMY.

Docent Dr. St. Szcz. Zaleski, of Dorpat, says that four or five years ago, while working in the Chemical Institute of the University of Dorpat, the assistant in the surgical clinic came with the remains of a silver tracheotomy tube, which had been left in for about two years without having been withdrawn or cleansed. Of the tube there only remained, owing to the solvent action of the contents of the air passages, the merest shell, in appearance like a kind of coarse cobweb. Thin, rough, uneven, sharp-edged portions were connected by silver threads, and it was surprising that the whole had hung together as well as it had done, and that a tube so nearly destroyed had not given way and caused the patient to die from asphyxia, or from injury of the air passages. The silver had been dissolved by the secretions of the bronchi and trachea, and had the tube been allowed to remain much longer, there can be but little doubt that no part of it would have been left.

He explains the chemical process by which the metal was dissolved by the continual action of the chlorine that exists in almost all the secretions of the body, and that is at times abundantly present in the mucous glands, upon the metallic silver which thus becomes converted into chloride of silver, this in its turn, although a very stable substance, being acted upon and dissolved by the alkaline secretions, which contain ammonia and cyanides, in the same way as the sulpho-cyanide of potassium of the saliva has the power of dissolving chloride of silver. This dissolved silver is of course partly expectorated, but part probably is absorbed. It may be that it goes directly to form organic compounds, or these may be reached by indirect means.

He does not know whether this patient suffered from argyria, but as soon as possible afterwards he performed tracheotomy on a cat, and inserted a silver biliary fistula tube, which was allowed to remain until the animal died a month later. By carefully weighing the tube before and after the operation, he found that it had sustained a very appreciable loss, the original weight, which was 1.9892 gramme, having been reduced by 0.0056 gramme, so that it had lost 0.282 per cent. of its total weight. It is thus quite evident that not only silver pessaries in the vagina, or silver tubes in biliary or gastric fistulæ, but silver tracheotomy tubes in the trachea, can be dissolved, the metallic silver being taken up by the fluids of the body.

Two questions present themselves: (1) To what extent is such a gradual wearing away of silver detrimental to the organism; and (2) what substance can best be substituted for silver in the construction of tracheotomy tubes? As regards the first question, we know that the long-continued and constant internal, and even sometimes external, use of silver preparations in very small doses ultimately produces argyria, which, as both clinical experience and experiments on animals teaches us, is a very serious and incurable disease. In the well-known case published by Orfila (who first described argyria), the use of nitrate of silver for eighteen months was followed, not by argyria merely, but by death.

In the case described by Fromman the gray discoloration of the skin occurred after ten months' treatment, during which period the patient had taken altogether 104.51 grammes of the nitrate, equivalent to 66.39 grammes of metallic silver. Riemer also published a case in which the patient became affected after thirteen months' treatment. During this period, he had taken 2,900 pills containing 17.40 grammes of the nitrate, or 21.61 grammes of metallic silver; death following after the consumption of 5,672 pills, which contained 34.032 grammes of nitrate, or 21.61 grammes of metallic siver. Finally Dittrich published a case in which toxic symptoms came on after six months' treatment of a tabetic patient, after he had taken 1.83 grammes of the nitrate (10 milligrammes daily), or 1.16 grammes of metallic silver, the patient succumbing at the end of two years of this treatment after having taken 7.32 grammes of the nitrate—equivalent to 4.69 grammes of metallic silver.

It will thus be seen that the manifestation of the deposition of silver in the tissues by the occurrence of a gray discoloration of the skin takes some time, but the actual deposition of the metal in the liver, bone, medulla, kidneys, and spleen commences very much sooner, producing what has been called *latent argyria*, during which there is no discoloration, though the health is affected, as experiments on animals show.

When it is considered that a silver tracheotomy tube weighs from 10 to 16 grammes, and that, if only half of it is dissolved, from 5 to 8 grammes are absorbed into the tissues, we must admit that all the conditions are present for the production of argyria. Of course, a certain part is expectorated, but by far the larger part is absorbed, at all events, as soon as the mucous membrane of the air passages has become inflamed. If we look upon argyria as a grave and dangerous affection, as it is considered by the majority of physicians, the conclusion is forced upon us that the use of such a comparatively easy soluble metal as silver for the construction of tracheotomy tubes is fraught with danger to life. Again, the danger of an irritant action of the dissolved silver on so delicate a tissue as the mucous membrane of the air passages, setting up a catarrhal condition, should not be lost sight of.

There is also another circumstance that must be taken into account in estimating the sources of danger. If the patient or his attendants are so negligent that the tube is not cleaned and examined as to its perfect condition, the destructive action will go on until a time comes when, as in the case related above, it will have become thin, and only threads and films of metal remain—a condition that renders the patient's life most precarious, as there is always grave danger of his being choked or injured by fragments broken off. That there is a possibility of this happening cannot be doubted after the case mentioned.

Regarding the second question — of what material the tube should be constructed in order that it may remain for a long time in the trachea without danger—for patients in good circumstances the most suitable materials are gold, platinum, or rock crystal. For those in the humbler classes, porcelain, glass, or even ivory might be used. These substances are, it is true, to some extent acted upon by the fluids of the body, the silica of glass being even soluble in traces in distilled water, but not in any appreciable quantity; besides which silica is one of the substances which is contained in the body, especially in the case of vegetable eaters, and is not hurtful to it. Gold and platinum may doubtless, after a long time, be to some extent dissolved, especially by the sulphuretted hydrogen, that if the disinfection is not perfect, is sure to exist.—*Lancet*, April 28, 1888.

---

### CARDIAC AND ARTERIAL AFFECTIONS AFTER TYPHOID FEVER.

The cardiac and arterial lesions found at the autopsies on typhoid subjects, says M. L. H. Petit, though they may have caused no symptoms during life, show that they do arise in the course of the disease, and render it impossible for the physician to ignore the relation between the cause and the effects; though the period of latency that separates typhoid fever from the cardiac affection masks to some extent this relation. Examination of these cardiac lesions shows that the orifices of the heart are rarely altered, and that the lesions especially affect the muscular structure of the heart, the right heart being almost sound in all cases.

The aorta and the large branches of the aorta show more evident alterations; the aorta is usually dilated, its external surface injected, its internal surface only a little affected, though often the seat of slightly projecting and irregularly distributed milky plaques.

The microscope shows that the muscular alterations are situated especially near the apex and the columnæ of the mitral valve, an important fact by reason of the effect on the functionation of the valves. The muscular fibre undergoes either granulo-fatty degeneration, or atrophy caused by compression resulting from the acute proliferation of the interstitial tissue. The arteries are attacked in all their parts by a sort of vegetation that causes obliteration of the cardiac arterioles. The most important lesion seems to exist in the muscular fibres; it consists of an active proliferation of the embryonal cells and of a sort of acute œdema that dissociates the muscular elements by creating shall lacunæ that give a sponge-like appearance. The hyperplasia of the connective tissue, characterized by the afflux of migrating cells and by proliferation in the place of the fixed cells of the connective tissue, contributes to the strangulation of the muscular elements, which promptly undergo simple atrophy or granulo-fatty degeneration.

These angio-cardiac lesions may be recognized for a longer or shorter time after an attack of typhoid fever, for months, or even for years, during which time the lesions organize and modify little by little the functionation of the circulatory apparatus by changing the normal state to a more or less pronounced pathological one.

From a clinical point of view the cardio-vascular manifestations of typhoid fever are seen in two different aspects; sometimes there is prompt generalization of the lesions, rapid enfeeblement of the heart or sudden arrest of it, giving rise to complications that are almost surely fatal; again, the cardiac or arterial alterations come on slowly, and in a shorter or longer time cause cardiopathies of variable intensity.

The first of these forms has been known for a long time. The second, to which attention was first specially called by MM. Landouzy and Siredy, comprehends the cardio-vascular affections that, coming on slowing and silently, and without marked symptoms in the course of typhoid, pass to the chronic state and make patients that are really cured of the typhoid fever veritable angio-cardiacs. The beginning is slow, and the cardiac affection is manifested only when the work of sclerosis is already advanced in the arteries or between the fasciæ of the myocardium. By reason of of the integrity of the orifices up to this time one cannot rely upon the evidence furnished by auscultation. It is therefore necessary to observe with care typhoid patients during the whole course of the disease, and during all the convalescence. To minute auscultation must be added careful examination of the pulse, examination at the apex to detect the least tendency to hypertrophy; the slightest peripheric troubles of the circulation must be attended to, and the urine should be minutely examined both as regards quantity and quality.

The first bruits that reveal typhoid lesions are constant neither as regards their situation nor their relation with the cardiac revolution; they may be heard over the mitral or aortic orifice, over the aortic sinus, and they may vary with the patient, and present in each one differences that are marked from day to day, according to the evolution of the cardio-aortic complication.

To these phenomena must be added pallor of the face, feebleness, slight dyspnœa, retro-sternal pains with irradiations toward the intercostal spaces of the left side, and of variable intensity, from a simple stitch up to the characteristic pains of angina pectoris; in the last case hypertrophy of the heart is easily made out. The aortic pulse appears at the same time.

The diagnosis of the cardio-vascular lesions of typhoid is easier than one would think, say Landouzy and Siredy. During typhoid and its convalescence minute auscultation, and daily examination of the pulse give the diagnostic signs. According to these authors the evidences furnished by the pulse are superior to those given by the temperature.

In the treatment of the primary arteritis the physician must resort to peri-cardiac and peri-aortic revulsion, under the form of the hot iron and cauterants, and the iodide treatment with caffeine will be of great service. The knowledge of the relations between typhoid fever and such cardiac and vascular lesions as have been spoken of will be of service in prophylaxis. —*L'Union Médicale*, No. 50.

### THE EYES IN CHOREA OF CHILDHOOD.

Dr. G. E. De Schweinitz, of Philadelphia, has just published in the *New York Medical Journal*, June 28, 1888, an account of the examination of the eyes of fifty cases of chorea of childhood. He draws the following conclusions:

1. The irides of choreic children quite commonly present chromatic asymmetry in shade, just as the same condition has been found in other forms of nervous disorders.

2. Slight differences in the width of the pupils may be found, but not more frequently—in fact, not as frequently—as these have been noted in perfectly healthy individuals.

3. Facial asymmetery is present in about one-half of the cases, just as this is present in cases of high refractive error, and also in individuals perfectly free from nervous disorders.

4. Hypermetropia and hypermetropic astigmatism are vastly the preponderating conditions of refraction in the eyes of choreic children, being found in about 77 per cent. of the cases, exactly as hypermetropic refraction is the preponderating condition in childhood generally, being found in 70 per cent. of the eyes of children in the elementary school years.

5. Imperfect equipoise of the eye-muscles is found in the great majority of the cases, but imperfect equipoise of the eye-muscles is very frequently present, in the eyes of school children free from chorea or neuropathic tendencies.

6. Embolism, atrophy of the disc, and optic neuritis may occur during or after attacks of chorea, but appearances in the fundus oculi characteristic of the disease have not been found.

7. As Octavius Sturges remarks: "It seems certain that a fairly constant proportion of chorea is directly connected with what may be called injudicious schooling, . . . but such nice adjustment as shall prevent overstraining on the one hand and overindulgence on the other is practically unattainable." Certainly an endeavor to lessen the overstrain of the eyes should be made. Hence the refraction errors and muscular defects in these children should be carefully and fully corrected by glasses, by prisms when necessary, or even by judicious surgical interference, and thus a probable exciting element removed; just as we should perform the same service for eyes similarly afflicted in children who are not choreic; just as we should improve the hygeine, remove the anæmia, treat the disabled circulatory apparatus in children who are not choreic. Evidence, however, seems quite as lacking that hypermetropic refraction is the basal cause of chorea as it is that chorea is the cause of the hypermetropia.

---

### SUBPERIOSTEAL REMOVAL OF HALF OF LOWER JAW; RECOVERY, WITH ALMOST ENTIRE RESTORATION OF THE BONE AND PERFECT MASTICATORY MOVEMENT.

Florence W., aged 6, was admitted to Charing Cross Hospital, on January 21, 1887, with extensive necrosis of the left half of the lower jaw. She had already been operated upon, and excision of the entire bone from the outside advised by a hospital surgeon. This the mother would not consent to; and she was placed under Mr. Bellamy's care.

On admission, the child had a most marked strumous aspect, the left side of the face being enormously swollen, the swelling extending over the temporal region. There was a sinus just in front of the external auditory meatus and one below the angle of the jaw.

On January 30, Mr. Bellamy enlarged the opening in the superior sinus, and removed the condyle, ramus, and angle, and opened up the remains of the sinus just under the angle. The periosteum was free and loose and retained the original form of the bone in a most remarkable manner.

February 1. The discharge was profuse and very offensive, although the cavity had been drained antiseptically. On the 7th the swelling began to decrease, and the discharge was quite healthy. On the 14th a small spicula of bone was found loose and removed from near the symphysis.

The child made an uninterrupted recovery, with the exception of some slight paralysis of the orbiculares pelpabræ and oris.

Seen on April 24, 1888. The two sides of the face are nearly equal in size; there is a small cicatrix behind the angle of the jaw; and on manipulation the new jaw feels somewhat nodulated, no doubt soon to mold itself into proper form. The articulation seems to be perfect, and the movements of the jaw as free as possible. In fact, there is no deformity at all. The teeth behind the first bicuspid have gone.—*British Medical Journal.*

### ANTIPYRIN IN POLYURIA AND DIABETES.

At a recent meeting of the Société de Thérapeutique de Paris, M. Huchard spoke of a case of excessive polyuria under his care, which had been benefited by antipyrin. The urine had decreased from 28 to 3 litres a day. At different times there was an increase when the drug was discontinued. At the time of the report none had been taken for several days, and there was no return of the polyuria. It is probable in this case, says Huchard, that the drug acted as a moderator of bulbo-medullar excitability. In this case also a violent attack of dyspnœa, which lasted two hours, was calmed by antipyrin. The substitution of ergot for antipyrin in this case caused an enormous increase in the urine. Bicarbonate of soda was then given with the antipyrin, and the urine rapidly decreased. The soda was at first given in doses of 2 grammes a day, and increased to 6 grammes.

In a second case, the patient passing about 10 litres a day, the urine was diminished one-half in five days. A third patient passed 800 grammes of sugar a day. In four days under antipyrin the urine fell from 12 to 3.90 litres after the administration of 6 grammes of the drug, and the sugar decreased to 71 grammes a day.

With two grammes of antipyrin Dujardin-Beaumetz obtained results parallel to those of M. Huchard. Albert Robin has seen the total disappearance of diabetes, without special régime, after the use of 4 grammes of the drug, in from 15 days to 3 weeks; but after a time the patients became albuminuric. It is known, however, that diabetics sometimes have intermittent albuminuria, and it is no cause for alarm when albumen appears temporarily in the urine of these patients.—*Le Bulletin Médical*, No. 30, 1888.

### A MAN WITHOUT A LARYNX.

The Paris correspondent of the *Lancet* writes: A short time ago I read a note in a scientific journal, entitled as above, which was reproduced from the Paris *Figaro*, and which was to the following effect: At No. 22, Rue de la Banque, Paris, may be seen a man (a wine merchant) who has been living without a larynx for the last two years. In April, 1885, he consulted Dr. Fauvel, the celebrated laryngologist, on account of severe dyspnœa, to which he had been subject for some time. Dr. Fauvel diagnosed an osteosis with œdema and ulceration of the larynx, and proposed to perform tracheotomy on the man. This he refused to submit to, and consulted other physicians; but, as he was getting worse, and the danger of suffocation became imminent, he decided upon the operation. Tracheotomy was practiced on February 13, 1886; but as this did not afford much relief, Dr. Péan, on February 27—that is, a fortnight afterward—performed ablation of the larynx This was done at the hospital, and on March 19 he was able to eat, and left the hospital cured and apparently in good health. I saw the man in his shop (a public-house) a short time ago, serving his customers as if nothing had happened to him. He is thirty-seven years of age, but looks about fifty. At my request he very willingly showed me his throat, where I perceived the cannula introduced by Drs. Péan and Fauvel, and whenever he had to answer my questions he closed the orifice of the cannula with his finger, and although his voice was not very audible, yet he spoke distinctly. He has not changed his habits, but eats and drinks as much as he ever did. As far as I could learn, the man had no antecedents of syphilis or tubercolosis, but had been frequently treated for "colds on the chest."

### LEPROSY IN RUSSIA.

The latest advices to hand point to the fact that in Russia, chiefly in the Baltic provinces, leprosy is increasing. In Lithuania, for example, there are to be found from two hundred and fifty to three hundred lepers, and in the district of Dorpat, it is said, the proportion of lepers is about one to a hundred of the population. There are reasons, however, for believing that the latter estimate is somewhat of an exaggeration. The inhabitants of these districts are naturally exhibiting some uneasiness in relation to the increase of this terrible disease in their midst, and have petitioned the government to appoint a commission for the purpose of inquiring into the facts, with a view to the adoption of some means for the arresting the propagation of the malady. It has just been decided to open a hospital at Riga with forty beds, for lepers, and this is undoubtedly a step in the right direction, though perhaps a small beginning. In Norway, where leprosy is also an epidemic, ample hospital accommodation exists for the treatment and isolation of the disease. Three leper hospitals are maintained by the State; one at Christiania in the south, another at Bergen in the west, and a third at Trondhiem in the north, into which the patients are admitted from all parts of the country. At any time during the course of the treatment the patients are free to return to their homes, in accordance with the belief in Norway, established by law, that the disease is not contagious.—*Medical Press and Circular.*

### BACTERIOLOGICAL EXAMINATION OF THE UTERUS AFTER PHYSIOLOGICAL PARTURITION.

Drs. Straus and Sanchez, Toledo, have made a number of researches and experiments on the females of certain animals with regard to the state of the uterine cavity after normal parturition. The first part of these researches consisted in bacteriological examinations of the uterine cavity and of the liquid it contained at different periods after parturition. The experiments were made on rabbits, guinea-pigs, and rats and mice. The animals were killed within from three hours to three days after parturition, and immediately after death the uterus and the Fallopian tubes were opened with all precautions.

The experiments showed that in these animals, after normal parturition, neither the uterine wall nor the uterine secretions contained any micro-organisms at all; and that the numerous germs that are found in the vagina do not penetrate into the uterus, or if they do are immediately destroyed. These experimental facts are analogous to those found by Döderlein in experiments on the lochia of the parturient woman: that the lochia of women having no fever do not contain bacteria, and do not produce toxic effects when inoculated on animals; while the lochia of women that have fever contain microbes, and do cause toxic symptoms when inoculated on animals.

Attempts were made also to inoculate these animals with pure cultures of different pathogenic microbes (bacillus anthracis, vibrion septicus, staphylococcus pyogenes aureus, and bacillus of chicken cholera). The last microbe was the only one that had any effect. There is thus seen to be a strange contrast between the resistance of the animal uterus and the vulnerability of the human uterus to pathogenic microbes.—*Gazette de Gynécologie*, May 15, 1888.

---

### RECENT OBSERVATIONS ON TUBERCULOSIS.

The stimulus given by Koch's discovery of the tubercle bacillus to a closer clinical observation of tubercular affections has resulted in a clearer understanding of some of the exceptional manifestations of tubercular disease. One of the most interesting additions to our knowledge in this respect is the proof, which is being continually strengthened by fresh observation, that the so-called dissection anatomical, or *post mortem* tubercle is really a manifestation of local tuberculosis, acquired by inoculation. Finger has lately published a case in which a man who had tubercular disease had suffered from five of these dissection warts which preceded the development of general tuberculosis, and in which microscopical examination showed the tubercle bacillus. Steinthal (*Monat. f. Prakt. Derm.*, 1888, p. 437) relates the case of a woman who was inoculated with tubercle on the skin of the hand through washing the clothes of her phthisical husband. Meyer (*Ibid*, p. 283) has related a case in which a child was infected with local tuberculosis of the genital organs by the wound made in the rite of circumcision being sucked by the operator, who was the victim of tubercular disease; and at a recent meeting of the Medical Society at Copenhagen, Dr. Salomon, sen., stated that he also had observed cases of tubercular infection produced in the same way by ritual circumcisers who practiced suction. On account of the dangers of the transmission of syphilis and tubercle in circumcision, Dr. Salomon, sen., advocated that the rite should be performed under precautions laid down by the authorities.—*British Medical Journal.*

---

### TARSECTOMY FOR SENILE ECTROPION.

At the recent meeting of the Société Française d'Ophthalmologie, M. Boucheron, of Paris, described under the name "tarsectomy" an operation for senile ectropion in the early stage. He practices excision of the tarsal cartilage, on the conjunctival side, when the ectropion is situated on the lower lid. The tarsectomy is partial when the ectropion is partial; and it is almost total when the ectropion is complete—that is to say, when the cartilage is totally luxated, and when the contracture of the orbicularis palpebrarum, in occlusion of the lids, maintains the tarsal luxation. Ablation of the tarsal cartilage, save the band one mm. broad close to the lids, pemits the orbicular muscle to go up to the brows, and the re-application of the lid to the eye.

The *redressement* of the lid is immediate. The permanence of the *redressement* is finally maintained by the formation of a small cicatricial conjunctival band substituted for the removed portion of the tarsal cartilage. The cicatrix, however, is of little importance, because the conjunctiva of the cul-de-sac unites with the ciliary border of the lid, either by first intention, or very rapidly by second intention.

There is no consecutive ectropion. A dozen operations have been made by Boucheron with perfectly satisfactory results.

While Boucheron does not insist so much upon the absolute novelty of the operation, he particularly insists on the facility, the security, and the value of it as he practices it: Application of Desmarres' or Snellen's forceps; incision along the orbital border of the tarsal cartilage; dissection of the cartilage; section and ablation of the cartilage one mm. from the free border; for partial ectropion, extraction in the same manner of the luxated part of the cartilage; compress dressings over the closed lids.—*Progrès Médical*, No. 19, 1888.

---

### ON THE TREATMENT OF SEBACEOUS TUMORS.

Many people, the subjects of congenital sebaceous tumors and "wens," object to having them removed, on the score that the remedy is worse than the disease, and the after-consequences may be serious.

The following is the method I have adopted in such cases, and with marked success. With a cataract knife (Graefe's) puncture the cyst, and gently squeeze out the contents; then introduce a very small piece of nitrate of silver. On the following day, by means of a pair of forceps, the capsule of the cyst can be withdrawn, just like the shell of a bean, without any portion being left adherent. In no case has there ever been any return of the growth or any ill effects.

The method, if tried, will be found to have many advantages apart from its simplicity and thoroughness.—*British Medical Journal.*

